# Sonic Hedgehog is expressed by hilar mossy cells and regulates cellular survival and neurogenesis in the adult hippocampus

**DOI:** 10.1038/s41598-019-53192-4

**Published:** 2019-11-22

**Authors:** Luis E. Gonzalez-Reyes, Chia-Chu Chiang, Mingming Zhang, Joshua Johnson, Manuel Arrillaga-Tamez, Nicholas H. Couturier, Neha Reddy, Lev Starikov, Jeffrey R. Capadona, Andreas H. Kottmann, Dominique M. Durand

**Affiliations:** 1 Neural Engineering Center, Department of Biomedical Engineering, Case Western Reserve University, Cleveland, Ohio 44106 USA; 2 Department of Molecular, Cellular and Biomedical Sciences, CUNY School of Medicine at City College of New York and Graduate Center, City University of New York, New York, NY 10031 USA; 3 Advanced Platform Technology Center, L. Stokes Cleveland VA Medical Center, Rehab. R&D, 10701 East Blvd. Mail Stop 151 AW/APT, Cleveland, OH 44106 USA

**Keywords:** Epilepsy, Molecular medicine

## Abstract

Sonic hedgehog (*Shh*) is a multifunctional signaling protein governing pattern formation, proliferation and cell survival during embryogenesis. In the adult brain, *Shh* has neurotrophic function and is implicated in hippocampal neurogenesis but the cellular source of *Shh* in the hippocampus remains ill defined. Here, we utilize a gene expression tracer allele of *Shh* (*Shh*-*nlacZ*) which allowed the identification of a subpopulation of hilar neurons known as mossy cells (MCs) as a prominent and dynamic source of *Shh* within the dentate gyrus. AAV-Cre mediated ablation of *Shh* in the adult dentate gyrus led to a marked degeneration of MCs. Conversely, chemical stimulation of hippocampal neurons using the epileptogenic agent kainic acid (KA) increased the number of *Shh*^+^ MCs indicating that the expression of *Shh* by MCs confers a survival advantage during the response to excitotoxic insults. In addition, ablation of *Shh* in the adult dentate gyrus led to increased neural precursor cell proliferation and their migration into the subgranular cell layer demonstrating that MCs-generated *Shh* is a key modulator of hippocampal neurogenesis.

## Introduction

Graded Sonic Hedgehog (*Shh*) signaling is a crucial regulator of cell proliferation, cell fate determination and migration, leading to cell diversification and congruent growth during early nervous system development^1^. In the adult brain, the *Shh* receptor Patched (*Ptc*) is expressed by neural stem cells (“B”-cells) and rapidly amplifying (“C”-cells) within the neurogenic niches of the subventricular zone (SVZ, forebrain) and subgranular zone (SGZ, hippocampus)^2^. Consistently, *Shh* is critically involved in B-cell maintenance and -profileration and in C-cell fate determination in the neurogenic niches of the adult forebrain and the hippocampus^3–5^. Whether neurogenic activity is controlled by physiological needs remains an active area of research. Variable *Shh* signaling strength within the germinal niche can determine the rate of neurogenesis and the type of cells being produced^2^. A critical step in investigating whether neurogenic outcome could be adapted to need is therefore the identification of the cellular source of *Shh* and the determination whether *Shh* expression is variable. The relevant cellular source of *Shh* for adult hippocampal neurogenesis, however, remains ill defined.

*Shh* was found to be expressed in calretinin positive neurons (CR^+^) of the hilus in the dorsal DG but not in the ventral DG in the early post-natal brain at P15^6^. The deletion of Shh from these CR^+^ cells was associated with a significant decrease in proliferation and the number neuronal stem cells (NSCs)^6^. Whether these neurons express *Shh* in the adult hippocampus has not been studied. In contrast, immunohistochemical analysis has suggested that pyramidal neurons^7^ or astrocytes^8^ might express *Shh* in the adult hippocampus. However, the failure to detect *Shh* mRNA in the hippocampus by *in situ* hybridization early studies, led some authors to propose that *Shh* could originate outside of the hippocampus. Thus, the protein would be produced by neurons in the basal forebrain cholinergic nucleus VDB^9,10^ where *Shh* transcription is abundant and anterogradely transported to the SGZ via the fimbria–fornix pathway^3^.

The difficulties associated with the identification of *Shh* cellular sources in the hippocampus might stem from the fact that *Shh* is a secreted protein. The presence of axonal transport signals in the *Shh* mRNA and protein sequence^11^ and the release of *Shh* from axons as well as from the somato-dendritic compartment^12^, yielding low and difficult to detect concentrations of both *Shh* mRNA and protein in the soma of *Shh* producing neurons. Furthermore, the *Shh* protein may accumulate in target cells that could easily be misidentified as sources^12^. We therefore re-examined the expression of *Shh* within the hippocampus using a sensitive *Shh* gene expression tracer allele which marks nuclei of *Shh* expressing cells by nuclear targeted lacZ and allows selective identification of cells in which the *Shh* locus is transcriptionally active. This reporter was used previously to discover that mesencephalic dopamine neurons are a significant source of *Shh* throughout adulthood in the forebrain^13^.

Mossy cells (MCs) constitutes a major population of CR^+^ neurons in the dentate gyrus (DG) of the hippocampus^14^. Extensive research has been performed to characterize MCs, but many of their functional and morphological properties remain elusive^15^. MCs are usually described as glutamatergic neurons that may exert feed-forward inhibition onto granular cells (GC) through GABAergic neurons^16,17^. However, no consensus has been reached as to whether the net effect of mossy cells on GCs is excitatory or inhibitory^15,18,19^. Many investigators assume that thorny excrescences define MCs, but there are spiny hilar cells without thorns that have the same physiological characteristics as ‘thorny’ MCs. Furthermore, MCs vary in their expression of neurochemical markers such as calretinin which is expressed in ventral but not dorsal mossy cells in mice (for review^15^).

Mossy cells could be implicated in SGZ neurogenesis driving glutamate and GABA transmission at different phases of granular cell development, but few studies have investigated specific interactions between MCs and neurogenesis in the adult brain^15^. Recently, Yeh *et al*.^20^ reported that MCs may control NSC quiescence through glutamatergic and GABAergic signaling. However, the notion that MCs could deliver *Shh* onto the NSCs as a possible activity-dependent regulatory mechanism of neurogenesis has not been explored so far.

Using a *Shh-nlacZ* genetic reporter^13^ we demonstrate here that *Shh* is expressed by most hilar MCs in the adult brain of mice. We find that *Shh* is expressed by most MCs and that these cells co- express GABA and glutamatergic markers. *Shh* expression reduces excitotoxicity of MCs in response to kainate induced epilepsy. Conversely, genetic ablation of *Shh* from hilar cells results in decreased numbers of MCs but increased migration of newly born neuronal precursor cells into the granular cell layer. Together, our results suggest that *Shh* expression in adult MCs serves as a neuro-protectant for MCs, as a chemo attractant for immature neuronal precursor cells that ectopically migrate to the hilus to become CR^+^ cells during induced excitotoxicity, and as an inhibitor of neuronal cell fates that home to the granular cell layer.

## Results

### Calretinin expressing GABAergic neurons are the source of *Shh* in the hippocampus

**Figure 1:**
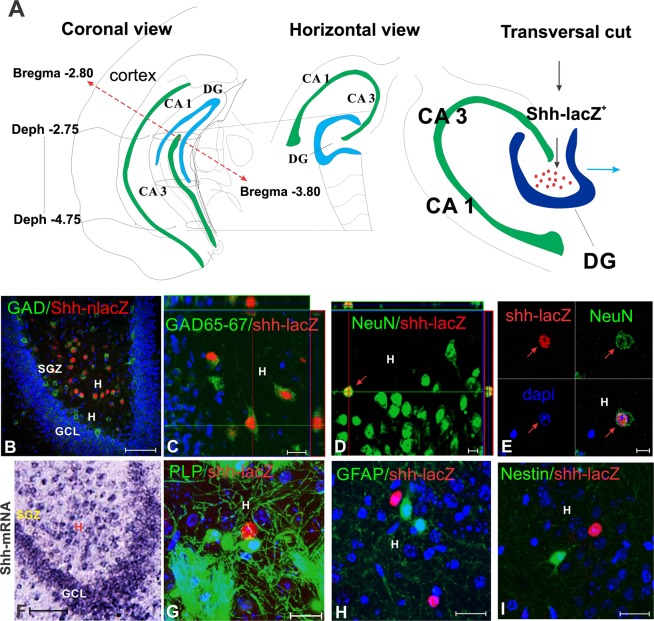
*Shh* is expressed locally in the hilus of the DG by GABAergic neurons in the adult brain (P60) (for quantification see Table 1). (**A**) From horizontal slices of the brain, we examine the whole ventral portion of the dentate gyrus. The horizontal brain slices yield transverse sections of the ventral portion of the hippocampus. (**B**) Low magnification confocal image of the hippocampus (transverse section) stained for GAD 65/67 and *Shh*-nlacZ showed that GABAergic neurons (stained for GAD 65/67) co-express *Shh*-*nlacZ* tracer. (25X, bar 100 µm). (**C**) Co-localization of *Shh*-*nlacZ* and GAD 65/67 at high magnification (100X, bar 20 µm) including an orthogonal view. (**D**) *Shh*-*nlacZ* positive cells were co-label with NeuN in the hilus near the granular cell layer (63X, bar 10 µm). (**E**) Confocal image of a cell located in the central field of the hilus on separated channels to show nuclear co-localization of *Shh*-*nlacZ* and NeuN (63X, bar 10 µm). (**F**) *In situ* hybridization of Shh in the ventral hilus at P56 cropped from Allen atlas row images (http://mouse.brainmap.org)^21^ (Bar 100 µm). (**G**) A double transgenic mice reporter for oligodendrocyte (*PLP*-GFP) and *Shh* (*Shh*-*nlacZ*) show that hilar oligodendrocytes did not express *nlacZ* (63X, bar 10 µm). (**H**) GFAP staining showed no co-localization with *Shh*-*nlacZ* indicating that *Shh* mRNA is not expressed in astrocytes in the hilus (63X, bar 25 µm). (**I**) Nestin staining showed no colocalization with *Shh*-*nlacZ* indicating that immature cells do not express *Shh* mRNA in the hilus (63X, 20 µm). *Abbr:* GCL, granular cell layer; SGZ subgranular zone; H, hilus.

**Figure 2:**
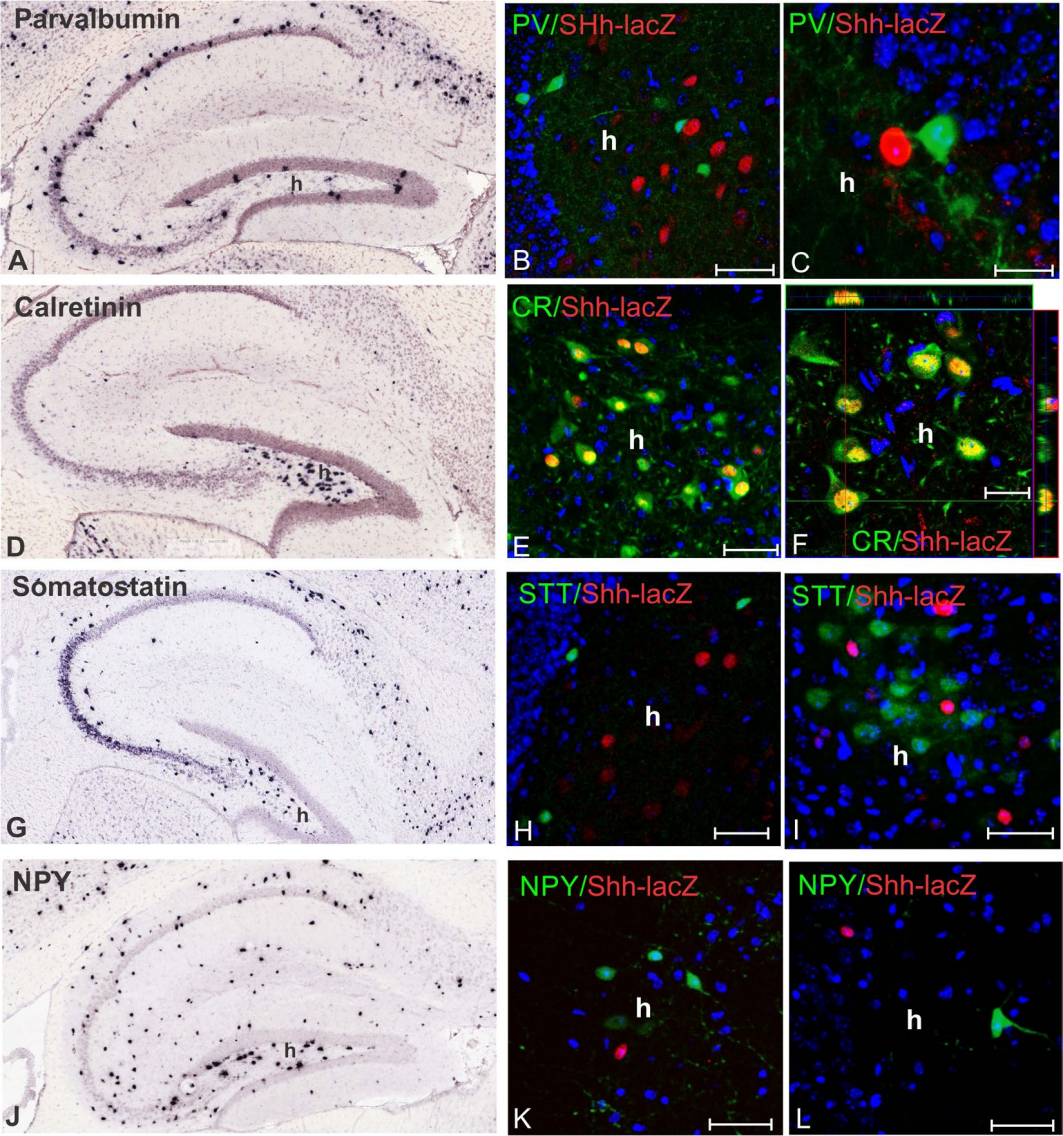
GABAergic neurons expressing *Shh*-*nlacZ*-mRNA are calretinin immunoreactive (for quantification see Table 1). (**A**) *In situ* hybridization in a control mouse showing parvalbumin expression pattern in the hippocampus; (**B,C**) Antibody staining for parvalbumin (PV) and *Shh*-*nlacZ* in the hilus. PV and *Shh*-nlcZ, show no colocalization (B, 25X, bar 50 µm; C, 63X, 20 µm). (**D**) *In situ* hybridization showing calretinin expression pattern in the hippocampus. (**E,F**) Immunostaining for calretinin and *Shh*-*nlacZ* in the hilus showing colocalization (E, 25, bar 50 µm; F, 63X, 20 µm). (**G**) *In situ* hybridization showing somatostatin expression pattern in the hippocampus. (**H,I**) Immunostaining for somatostatin and *Shh*-*nlacZ* in the hilus showing no colocalization (25X, bars 50 µm). (**J**) *In situ* hybridization showing NPY expression pattern in the hippocampus. (**K,L**) Immunostaining for NPY and *Shh*-*nlacZ* (see Table 1) in the hilus shows no colocalization. (K, 25X, bars 50 µm). *In situ* hybridization images were obtained from Allen Mouse Brain Atlas (http://mouse.brain-map.org)^21^*. Abbr*: h, hilus.

To identify the cells that produce *Shh* in the DG, we first visualized expression of *Shh* in the adult brain using mice homozygous for a gene expression tracer allele of *Shh* (*Shh*-*nlacZ*^+/+^)^13^. Here *Shh* and nucleus-targeted LacZ is transcribed into a bi-cistronic mRNA from the endogenous *Shh* locus such that all cells that express *Shh* are also marked by nuclear localized betaGal allowing sensitive chromogenic and fluorescent immunohistochemical analysis of *Shh* expression with single cell resolution. The use of anti-beta galactosidase antibodies in combination with cell type specific markers demonstrated that *Shh* is not produced by oligodendrocytes (*PLP*-EGFP, Fig. 1G and Table 1), astrocytes (GFAP, Fig. 1H and Table 1) or immature cells (Nestin, Fig. 1I and Table 1) but only by mature neurons (NeuN, Fig. 1D,E and Table 1). Most *nlacZ*^+^ neurons co-express GAD-65/67 (Fig. 1B,C, Table 1) and form a pattern similar to that seen by recent *in situ* hybridization for *Shh* (Fig. 1F, Allen Atlas^21^). We next investigated which sub-type of GABAergic neuron would express *Shh*. We stained brain sections from animals carrying the *Shh*-*nlacZ* allele with antibodies for parvalbumin (PV), somatostatin (STT), neuropeptide Y (NPY), and calretinin (CR), which are GABAergic neuronal subtypes present in the DG (Fig. 2) and as revealed by *in situ* hybridization (Allen Atlas^21^). We found that among all the cells that express the *Shh*-*nlacZ* tracer allele, 98.1 ± 8.4% co-expressed CR and, conversely, among cells expressing CR, 72.4 ± 3.9% expressed the *Shh*-*nlacZ* tracer (Fig. 2E,F, Table 1). The CR^+^ *Shh*-*nlacZ*^+^ cells exhibited a multipolar morphology containing large polygonal somata (diameter ≈ 20 μm) with abundant primary axodendritic arborizations forming a dense network within the hilus. We did not find any other GABAergic neuronal subtypes among the cells expressing *Shh*.

**Table 1:** Stereological analysis of *Shh* reporter expression in different cell populations in the dentate gyrus.

1.Label*	2.Cell Type	3.No. cells label	4.Label/LacZ	5.Colocalization % Label/LacZ	6.total LacZ	7.Colocalization %LacZ/Label	8.No. mice X slice
PLP	Oligodendrocytes	760 ± 38.9	0	0	n.q	0	3x10
GFAP	Astrocytes	1013.3 ± 83.7	0	0	n.q	0	7x10
NeuN	Neurons	3834 ± 320	1120 ± 51.1	29.2 ± 1.3	1138.2 ± 56	98.4 ± 5.0	3x10
CR	GABA subtype	1574.3 ± 62.4	1140 ± 62.4	72.4 ± 3.9	1162.1 ± 91.2	98.1 ± 8.4	7x10
SST	GABA subtype	1062.5 ± 113.3	0	0	n.q	0	3x10
PV	GABA subtype	528.6 ± 69.9	0	0	n.q	0	3x10
NPY	GABA subtype	462.5 ± 40	0	0	n.q	0	3x10
GAD 65/67	GABAergic	3433.3 ± 391.3	1114.3 ± 94.9	32.4 ± 2.8	1146.1 ± 81.3	97.2 ± 7.3	7x10
GlutR2/3	Glutamatergic	3116.7 ± 188.8	1300.2 ± 77.5	41.7 ± 5.0	1370 ± 87.9	92.4 ± 9.2	6X10

The *Shh*-lacZ reporter was expressed in only in neural phenotypes NeuN, GAD 65/67,CR and GluR2/3 and CGRP. This analysis leads to conclude that *Shh* is exclusively express in mossy cells that express CR and GAD. % Cells labeled for a cell type that express lacZ (column 5) and, conversely, lacZ^+^ cells that express a cell type label (column 7) were quantified. (n.q.): lacZ cells were “not quantified” (column 6) when the reporter was absent in the cell type of interest (column 4). The number of cells were estimated stereologically^13^ (see methods). *PLP (proteolipid protein), GFAP (glial fibrillary acidic protein), NeuN (neuronal nuclei), CR (calretinin), SST (somatostatin), PV (parvalbumin), NPY (neuropeptide Y), GAD (glutamic acid decarboxylase), GluR2/3 (Glutamatergic receptor 2 and 3).

### CR^+^ neurons that express *Shh* in the adult hippocampus are mossy cells (MC)

**Figure 3:**
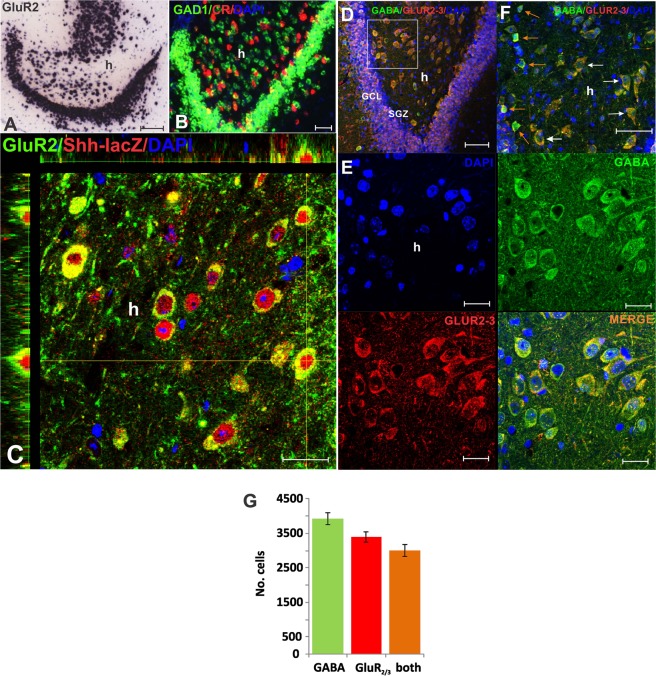
The Mossy cell identity is corroborated by reactivity to GluR2/3 antibodies. (**A**) Pattern of expression of GluR2 mRNA as revealed by *in situ* hybridization (http://mouse.brain-map.org)^21^, which resemble the expression of *Shh*-nlacZ and CR^+^ cells (bar 100 m) (mouse P57) (Bar = 100 µm). (**B**) Double fluorescent *in situ* hybridization for GAD1 and CR (http://mouse.brain-map.org)^21^ showing colocalization of the two mRNAs (CR/GAD-1 = 71.8 ± 5.2%; GAD-1/CR = 50.2 ± 2.4%,estimated from 2 mice, P56) (Bar = 45 µm). (**C**) IHC staining for GluR2/3 and *Shh*-nlacZ showing an orthogonal view to demonstrate colocalization (see Table 1, columm-7). (100X, Bar = 20 µm). For the cell numbers marked by GluR 2/3 and LacZ see Table 1. (**D**) Staining for GluR2/3 and GABA confirm the colocalization, revealing that mossy cells are immunoreactive to GABA. (20X, bar = 50 µm). (**E**) Zoom into D (100X) showing hilar neurons different channels confirming colocalization of GluR2/3 and GABA at high magnification (100X, bar = 20μm). (**F**) An additional double fluorescent IHC section that shows GABA and GluR2/3 colocalization, and high (orange arrow) and low (white arrow) levels of GABA staining in cell positive for both GluR2/3 and GABA or cells that are only immunoreactive to GABA (40X, bar = 50μm). (**G**) Analysis of the GABA and GluR2/3 co-expression show that a 76.5% of GABA^+^ cells colocalized with GluR2/3; while an 88.3% of GlutR2/3^+^ cells colocalized with GABA. (n = 3 mice × 10 slices). *Abbr:* GCL, granular cell layer; SGZ subgranular zone; and h, hilus.

We next determined whether the cell population described here were mossy cells (MCs), a prominent subpopulation of CR^+^ cells with elusive function. We found that cells expressing *Shh*-*nlacZ* co-localize with glutamate receptor 2/3 (GluR2/3) expression, a marker for MCs (Fig. 3C, Table 1). Among the GluR2/3+ neurons, 41.7% were *Shh*-*nlacZ*+ (Table 1, columm- 5), and among *Shh*-*nlacZ*+, 92.4% were Glut R 2/3+ (Table 1, column-7). Further, over 90% of the cells expressing *Shh*-*nlacZ* are labeled for both CR and GlutR2/3 markers (Table 1, column-7). Consistent with the immunohistochemical staining, the GluR2 mRNA *in situ* hybridization image cropped from the Allen atlas^21^ (Fig. 3A) resembles the distribution of *Shh*-*nlacZ*+ or CR+ cells in the hilus. Further, the majority of α-GluR2/3 stained soma colocalized with GABA in the hilus (Fig. 3D–F). Consistently, double-color fluorescence *in situ* hybridization (FISH) for CR (Calb2-IRES-Cre) and GAD1, show that most hilar CR^+^ cells (≈75%) express GAD-1 mRNA (Fig. 3B) (Allen Atlas^21^). Therefore, based on their distribution, morphologic features and staining for CR, GluR2/3, these results reveal that MCs remain as a prominent source of *Shh* in the adult hippocampus. The prevailing view is that CR and GluR2/3 are markers for mossy cells while MCs are thought to be non-immunoreactive to GABA markers. To further test the notion that MCs may express GABA, we decided to use another mossy cell-specific marker named calcitonin gene-related peptide (CGRP)^15,22,23^. We found that about 80% of the CGRP^+^ cells colocalize with GABA (Supplemental Fig. S2). As the three MC markers (CR, GluR2/3 and CGRP) highly colocalize with GABA, it follows that mossy cells also express GABA even if these cells are not functionally GABAergic (see below).

### Mossy cells that produce *Shh* are resistant to KA toxicity

**Figure 4:**
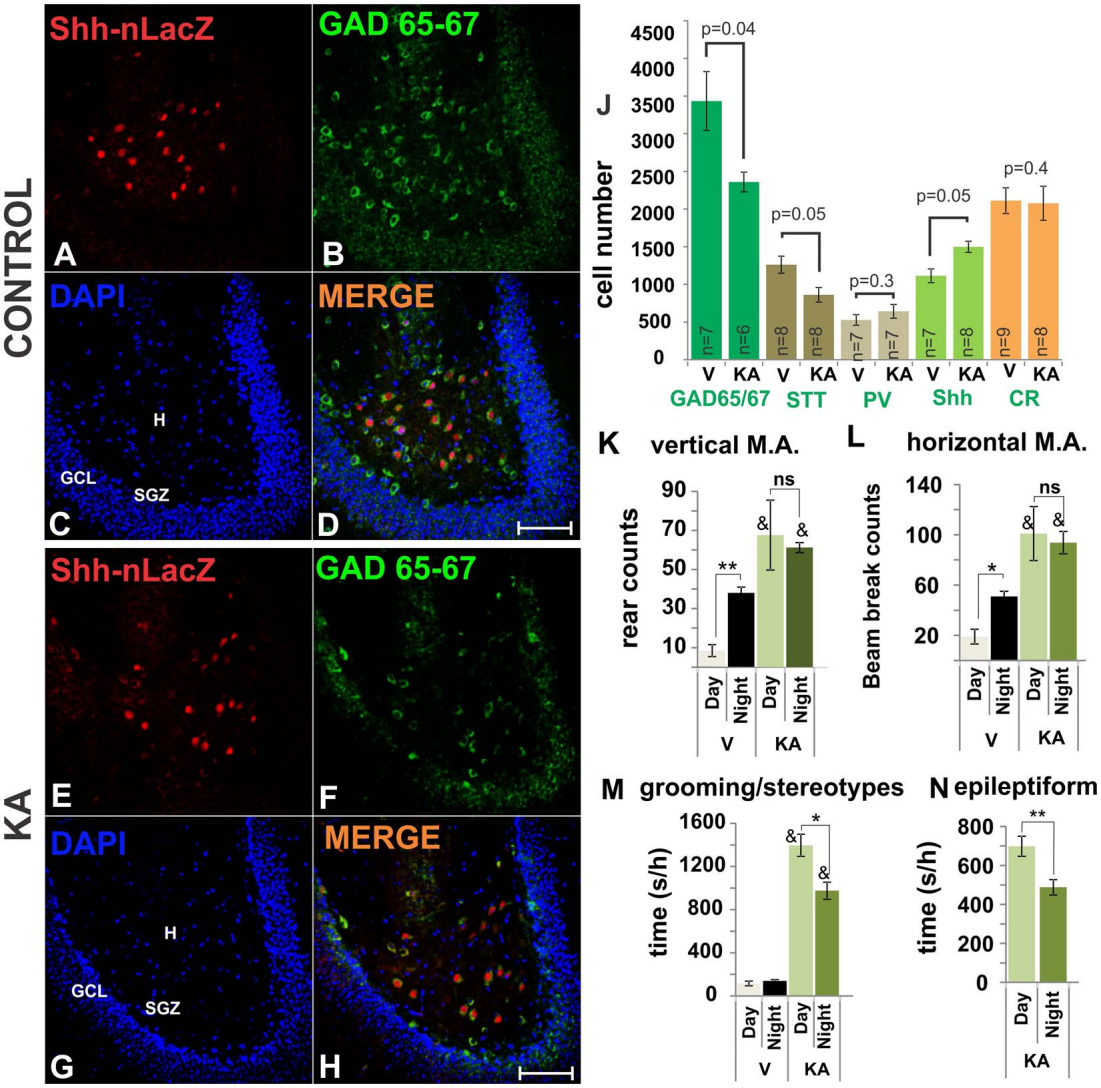
KA administration induces loss of large number of GABA cells but GABAergic Shh-*nlacZ*+ neurons seem unaffected. (**A–D**) Shh-*nlacZ*+ GAD 65/67+ neurons in the DG of control animals showing co-localization (25X, bar 100 µm). Note the full complement of cells expressing Shh-lacZ in the hilus (red channel). (**E–H**) The number of Shh-*nlacZ*+ neurons did not decrease while the number of GAD 65/67+ neurons appears to diminish at 2 weeks following KA injections (25X, bar 100 µm). (**J**) Cells expressing Shh-nlacZ were up-regulated, while the overall expression of GAD 65/67 decreased. GABAergic subtypes included PV, parvalbumin; SST, somatostatin; CR, calretinin and Shh, sonic hedgehog. Examples of these staining are provided in Fig. 2. (p values correspond to Unpaired Student’s t-Test). (**K–N**) Behavior was analyzed on the second week after injection, scores correspond to average counts/h or seconds/h of a given behavior for 7 days, 4 h/day(12–4 pm) and 4 h/night (12–4am). *p < 0.01, **p < 0.001, night vs. day; & p < 0.001 KA vs. vehicle group. Epileptiform activity was always equal or lower than stage 3 (mouse forelimb clonus without rearing) (n = 6/group). Abbr: GCL, granular cell layer; SGZ subgranular zone; H, hilus; M.A., motor activity.

We next sought to evaluate whether MCs expressing *Shh* were endowed with greater resistance to kainic acid (KA), a neurotoxic and epileptogenic agent, compared to GABAergic cells that do not express *Shh*. *Shh-nlacZ* mice were injected with increasing doses of KA (IP 5 mg/kg/h) to a maximum of 35 mg/kg until status epilepticus was reached (Racine’s stage 4/5)^24^. The behavioral assessment of the animals during the second week after KA injections revealed increased motor activity, exaggerated grooming, stereotypes and epileptiform jumping, shaking and forelimb clonus (Hyperexcitability or Racine’s stage 3) (Fig. 4K–N). These observations indicated that at this point the animals have not achieved full development of the epileptic phenotype but rather were engaged in an epileptogenic process. To characterize this stage, histological changes were studied in these animals and to avoid confounding effects derived of the stereotaxic surgery and electrode implantation, the electrophysiological effects of the KA injection were studied in a separated group of mice subject to the same procedure. The behavioral changes shown in Fig. 4 parallel the increased neural activity in the hippocampus as shown by EEG recordings (Supplemental Fig. S1). The EEG also showed typical interictal activity consistent in high-amplitude bursting and hyper-synchronized spiking in KA-injected animals, which are specific abnormalities associated with the epileptogenic stage.

Two weeks after KA injections animals were euthanized and their brains were processed for immunohistochemistry (IHC). We stained for *nlacZ* and GABAergic markers in the hippocampus. The level of *Shh* expression per cell as measured by the stained surface area and fluorescence intensity of the *nlacZ* immunostaining did not differ between the groups [surface area stained by *nlacZ* for vehicle, 63.2 ± 6.4 and for KA, 60.2 ± 5.5, T-test, p = 0.7; Normalized O.D for vehicle, 100 ± 9.5 and for KA, 97 ± 7.5, T-test, p = 0.4; based on 1029 cells (Veh) and 933 cells (KA), n = 3 mouse × 10 slices]. Interestingly, despite a decrease of about 40% (p = 0.04, Student’s t-test, Fig. 4H,J) of the numbers of GAD-65/67^+^ cells, the amount of GABA cells expressing *Shh*-*nlacZ* was *increased* by about 20% (p = 0.05, Student’s t-test, Fig. 4J) compared to controls. The total number of CR^+^ cells was unchanged in the KA treated animals. The increase in the prevalence of *Shh*+ cells among GABAergic neurons in the face of KA dependent neuronal degeneration suggests that *Shh* expressing cells are more resistant to excitotoxicity than *Shh* negative neurons, or that KA induces (1) the expression of *Shh* in previously *Shh* negative neurons or (2) the de novo differentiation of *Shh* expressing neurons.

**Figure 5:**
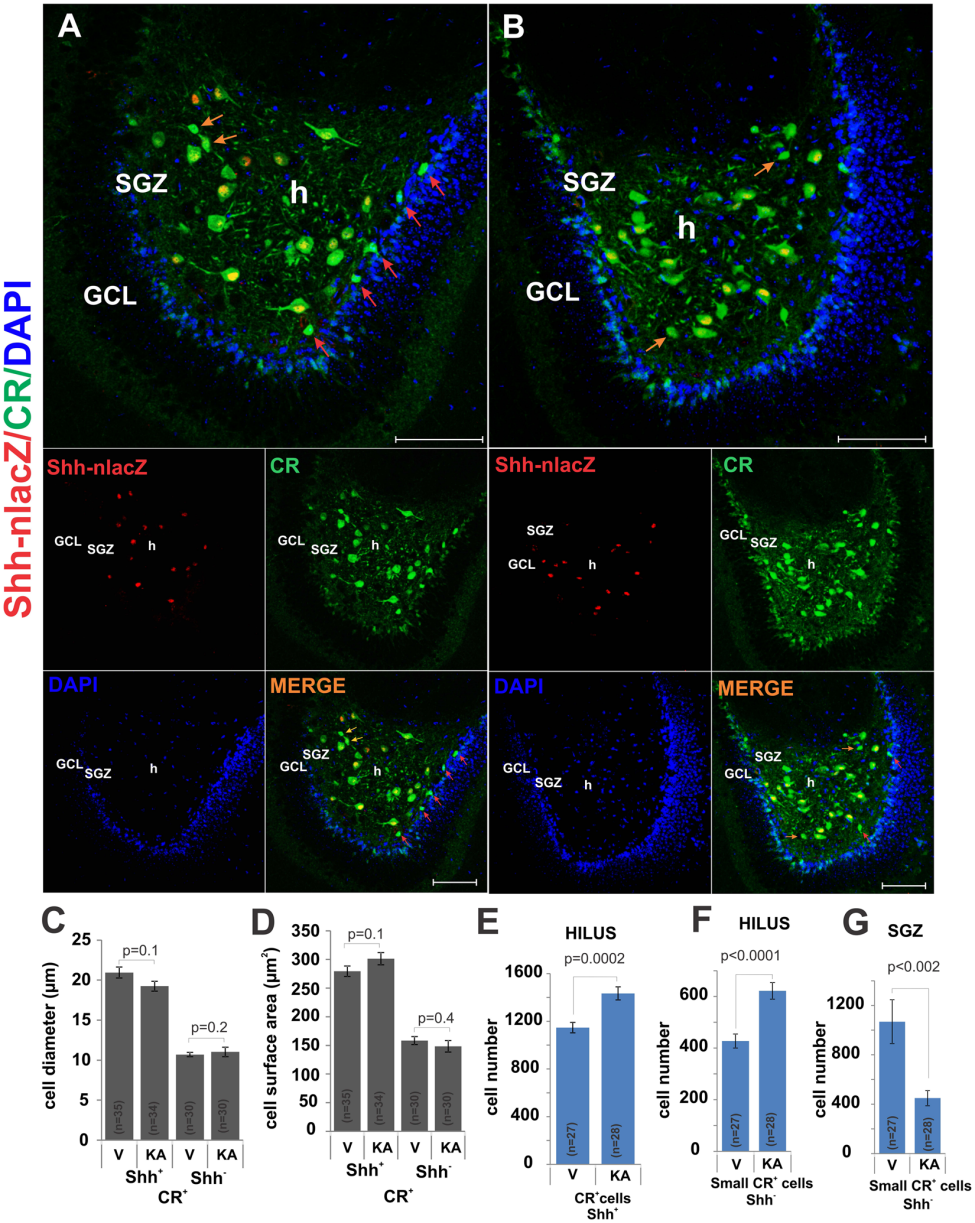
KA administration altered the proportion of CR+ cell subtypes in the SGZ and hilus. (**A**) [Control] Shh-*nlacZ*+ and CR+ neurons in the DG of control animals showing co-localization (25X, bar 100 µm). (**B**) [KA] The number of CR+ Shh-*nlacZ*+ neurons in the KA group seems to be unaffected relative to controls (25X, bar 100 µm). Note that the small CR+ cells in SGZ (red arrows in A) have decreased in the SGZ as compared with controls (A), while the number of small CR+ cells in the hilus (some example show by yellow arrows) appears to increase. (**C,D**) CR+ Shh-*nlacZ*+ neurons have greater diameter and surface area than CR+ Shh-nlacZ- neurons. These parameters were not affected by KA treatment. (**E,F**) The number of large CR+ Shh-*nlacZ*+ cells as well as small CR+ Shh-nlacZ- cells from the hilus increased following KA injection. (**G**) The number of small CR+ Shh-nlacZ- cells from SGZ decreased in the KA group. (p values correspond to Unpaired Student’s t-Test). Abbr: GCL, granular cell layer; SGZ subgranular zone; and h, hilus.

To distinguish these possibilities, we focused on CR^+^ cells and determined the morphological characteristics and locations of CR^+^*Shh*^−^ and CR^+^*Shh*^+^ neurons in the hippocampus of untreated animals. As quantified above, a minority of CR^+^ cells do not express *Shh* (Fig. 4J). These *Shh*^-^cells are located in the hilus where they make up 14% of all CR^+^ cells and in the SGZ where they make up 35% of all CR^+^ cells (Fig. 5). These two CR^+^*Shh*^−^ groups differ from *Shh*^+^CR^+^ neurons by morphology. CR^+^*Shh*^−^ neurons exhibited small round somata (diameter 10.7 ± 0.3 μm, surface area ≈ 150 μm^2^; Fig. 5C) and small dendritic arborizations, while the CR+ *Shh*^+^ neurons possessed a larger somata (diameter 20.9 ± 0.7 μm, surface area ≈300  μm^2^) and abundant primary axodendritic arborizations. KA treatment increased the number of both large CR^+^*Shh*^+^ multipolar neurons and small round CR^+^*Shh*^−^ cells in the hilus (Fig. 5A,B E,F) but decreased the number of small CR^+^*Shh*^−^ neurons in the SGZ (Fig. 5G). These findings are in line with previous results that revealed that the number of hilar CR^+^ cells increased 2.5 times in mice injected with intra-DG KA^25^.

**Figure 6:**
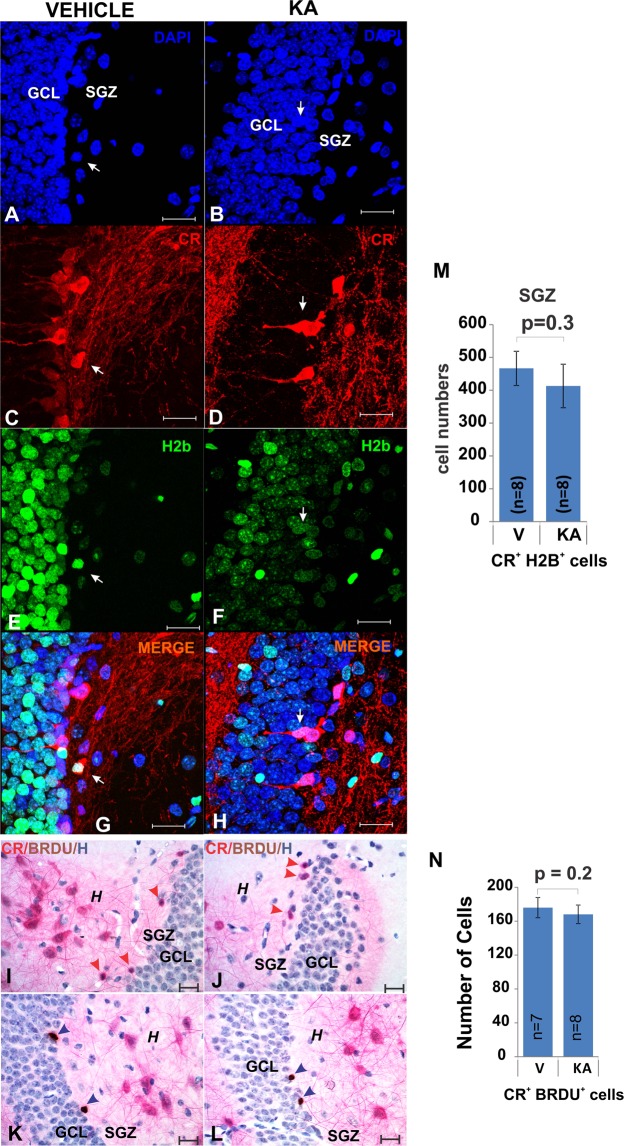
KA treatment did not affect SGZ proliferation. (**A–H**) and (**M**). The number of CR+ cells that co-localize with the H2B-eGFP (mitotic marker) (white arrows) nuclear signal did not increase in KA-injected animals at 2 weeks after KA injections (60X, bar 20 µm). (For H2B-eGFP see Methods-transgenic animals). (**I–J**) and (**N**). The number of small CR cells in the SGZ that co-express BRDU (which was injected 7 days before brain perfusion) did not differ between controls and KA-injected animals. Chromogenic staining using antibodies for calretinin and BRDU (hematoxylin as counterstain) showing examples of CR+ BRDU+ cells (red arrowheads) (newborn post-mitotic neurons in the SGZ) (60X, bar 20 µm). (**K**,**L**) The number of SGZ BRDU+ cells (BRDU was injected 7 days before brain perfusion) was not different between controls and KA-injected animals. The staining was the same as in I-J-above (60X, bar 20 µm). Abbr: GCL, granular cell layer; SGZ subgranular zone; and h, hilus.

We tested next whether the increase of CR^+^ cells in the hilus might have been caused by increased neurogenesis. In agreement with previous reports^26–28^, however, we did not find increased numbers of cells expressing the mitosis marker phosphorylated Histone 2B (pH2B) (Fig. 6A–H,M), or that had incorporated the nucleotide analog BrdU (Fig. 6I–L,N) in the SGZ suggesting that KA treatment did not induce an increase in the rate of neurogenesis. Therefore, in the absence of increased proliferation, our observations suggest that KA might induce increased migration of immature, small CR^+^ cells from the SGZ into the hilus where they mature into large CR^+^*Shh*^+^ neurons. This possibility is consistent with the previous finding of ectopic migration of newborn cells from the SGZ to the hilus in the KA model^29^ and with the increase in CR^+^ neurons following KA administration^25^.

### The *Shh* signaling effector Smoothened is not expressed by CR^+^ neurons under normal conditions

**Figure 7:**
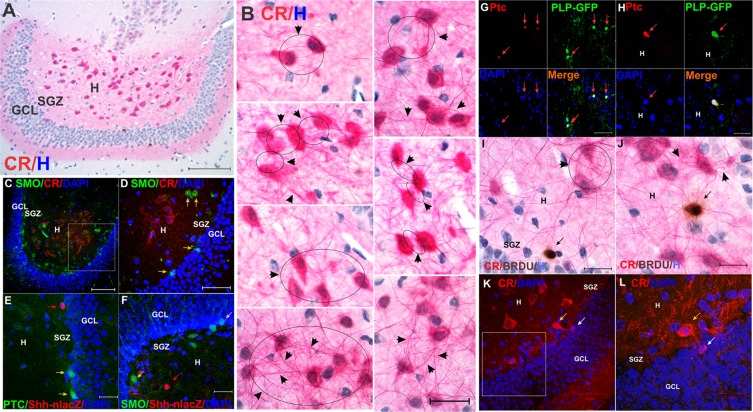
Pattern of reciprocal innervation of ventral DG CR+ cells and expression of Shh receptors. (For quantification see Table 1). (**A**) Panoramic view showing abundant numbers or large CR+ neurons in the ventral DG (10X, bar 100 µm). (**B**) Detailed view of CR cells and their axonal/dendritic processes, showing profuse reciprocal connectivity (arrow-circles and arrowheads), which is a typical feature of CR (60X, bar 50 µm). (**C**) Expression of Smo and CR is segregated in the hilus. Most CR+ cells are located in the central hilus, while Smo+ cells are located in the peripheral SGZ (25X, bar 100 µm). (**D**) Close-up of C showing Smo positive cells (yellow arrows) in the SGZ (25X, bar 50 µm). (**E**) Shh-nlacZ (red arrow) and Ptc-1 (yellow arrow) do not co-express in the DG (for quantification see Table 1) suggesting separated cellular sources (63X, 25 µm). (**F**) Shh-nlacZ (red arrow) and Smo (yellow arrow) do not co-express in the DG (for quantification see Table 1) suggesting independent cellular sources (63X, 25 µm). (**G**) Ptc-1 is expressed by olidodendrocytes (PLP/Shh-nlacZ, 92.1 ± 4.7%, n = 3 × 10 slices) that do not co-express Shh (see Fig. 1D) (40X, bar 50 µm). (**H**) Larger magnification as in G (63X, bar 20 µm). (**I**) Hilar CR+ projections innervating a BRDU^+^ progenitor (black arrow) in the SGZ. Immunohistochemistry was performed with chromogenic staining for CR (Vulcan Fast Red), BRDU (DAB) and hematoxylin (H) as counterstaining (60X, bar 20 µm) (arrowheads and circle also show reciprocal innervation). (**J**) Hilar CR+ projections innervating a BRDU^+^ progenitor (black arrow) located in the central hilus. Immunohistochemistry was performed with chromogenic staining as in I (60X, bar 20 µm). (arrowheads show reciprocal innervations as in I). (**K**) Example of a calretinin (CR^+^) neuron from the hilus (yellow arrow) innervating a SGZ CR+ neural progenitors (white arrow) (63X, bar 50 µm). This suggests that CR^+^ neurons from the hilus innervate CR+ SGZ newborn neurons. (**L**) Close-up of K showing innervation of a CR^+^ progenitor (white arrow) by a CR^+^ neuron (yellow arrow) (63X, bar 20 µm). Abbr: DG dentate gyrus; GCL, granular cell layer; SGZ subgranular zone.

*The Shh* receptor *Ptc* and the obligate necessary *Shh* signaling effector Smoothened (*Smo*) are expressed in the SGZ on neuronal progenitors^3,4^. Calretinin based chromogenic staining (Fig. 7) showed profuse reciprocal innervation between CR^+^ cells (Fig. 7B), as described previously by Gulyás *et al*.^30^, as well as CR^+^ innervation of both SGZ (Fig. 7I) and hilar progenitors^20^ (Fig. 7J). Interestingly, hilar CR^+^ neurons innervate immature CR^+^ cells in the SGZ (Fig. 7K-L). Because of this pattern of reciprocal innervation observed between CR^+^ neurons, we wanted to investigate whether hilar CR^+^ neurons were able to perceive *Shh* signaling and therefore might not only act as a source but also as a target for *Shh* signaling. Staining of CR^+^ cells in the hilus (Fig. 7C,D) for *Ptc* (Fig. 7E) and *Smo* (Fig. 7F) revealed no co-expression with *Shh-nlacZ*. In contrast, oligodendrocytes (*PLP*+ cells) did not express *Shh-nlacZ* (Table 1), but *Ptc* suggesting that these cells are likely local recipients of *Shh* signaling in the untreated brain (Fig. 7G,H). These results indicate a paracrine mode of *Shh* signaling that originates from hilar MCs in the untreated DG (Fig. 7, Table 1).

### KA alters the pattern of *Smo* expression in the hilus

**Figure 8:**
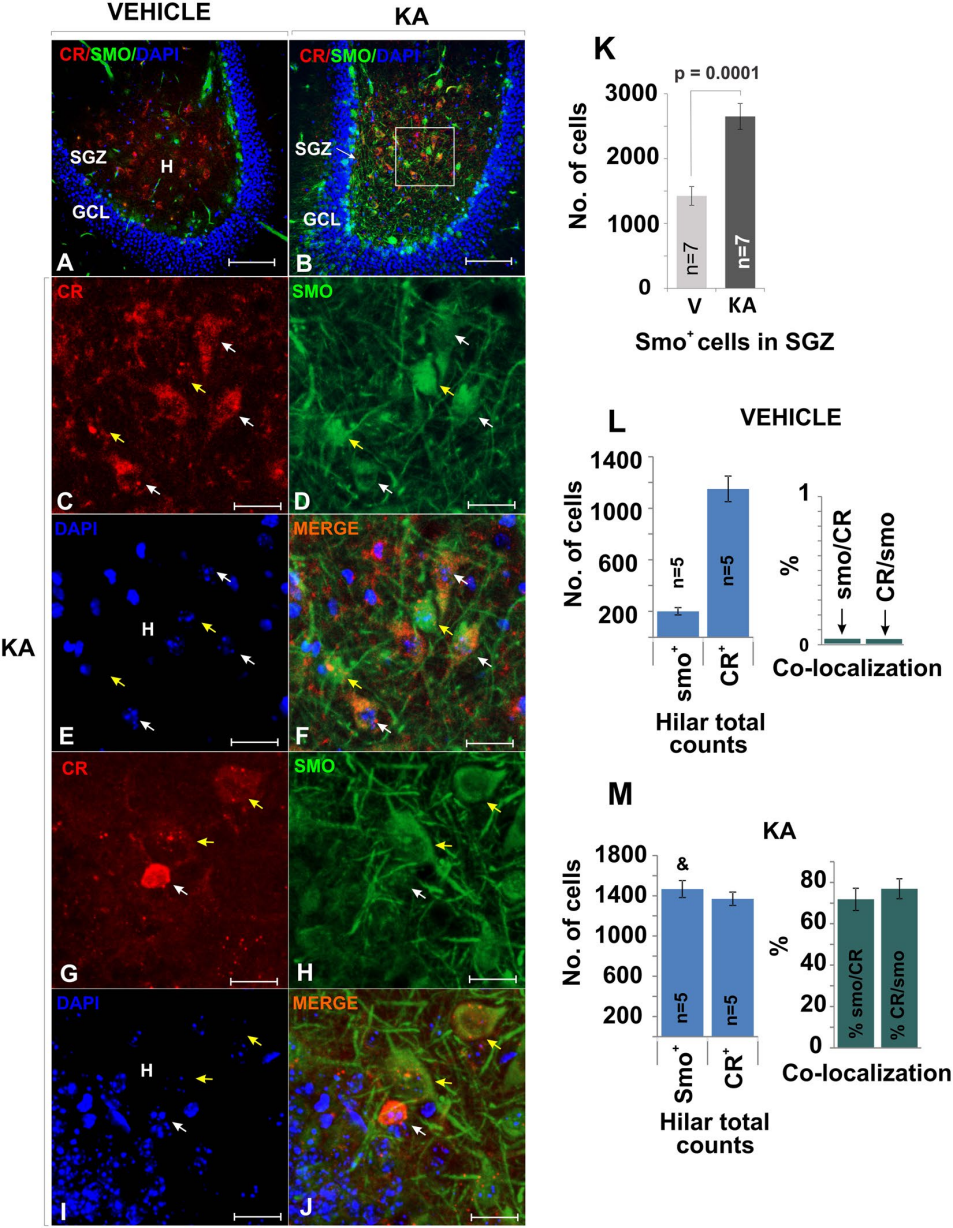
Smo is expressed by hilar CR+ cells following KA treatment. (**A,B**) The Shh downstream molecule Smo was up-regulated in animals that received KA injections. In A, smo is preferentially detected in the SGZ but in B Smo and CR staining colocalized in many cells in the central hilus. (25X, scale bar 100 µm). (**C–F**) High magnification as in B (63X, bar 25 µm). Example of cells that express smo but do not express CR (white arrow) and CR^+^ cells that express both Smo and CR (yellow arrow). (**G–J**) More examples at high mag (63X, bar 25 µm) of cells that expresses smo but does not express CR (white arrow) and cells that express both Smo and CR (yellow arrow). Note that the small CR^+^ cell showing high intensity for CR and very weak staining for Smo is an SGZ-immature neuron. (**K**) Smo was significantly up-regulated in the SGZ in animals receiving an epileptogenic dose of KA. (**L,M**) Both Smo-CR and CR-Smo co-localization increased from near 0% to about 70% in animals injected with KA. Quantification of cells in the hilus across the ventral hippocampus in vehicle treated mice show that Smo expression was relatively small (as in A, restricted to the SGZ). Smo and CR colocalization was not observed. The number of cells expressing Smo was significantly greater in KA treated mice (M) as compare to Vehicle (L)(&, KA vs. Control, p = 0.00001, student T-test), while CR^+^ cells number was unchanged. The co-localization of the two markers was higher in the hilus of KA treated mice (CR/Smo = 70%, Smo/CR = 70%). Abbr: GCL, granular cell layer; SGZ subgranular zone; and h, hilus.

Guided by results that revealed that the expression of *Smo* in the Hippocampus can be altered by electroconvulsive seizures^10^, we next tested whether KA treatment alters the expression of *Smo*. In vehicle treated animals CR and *Smo* did not co-localize (Figs 7 and 8A), but in the KA-treated animals *Smo* expression was upregulated in the hilus and SGZ (Fig. 8B–J,K) (*Smo*/CR, CR/*Smo* ≈70%) (Fig. 8M). These observations suggested that under KA-induced hyper-excitability, CR^+^ cells become receptive to *Shh* signaling, which may enhance the survival capacity of these CR^+^ cells.

### Selective ablation of *Shh* expression in CR^+^ neurons leads to CR cell loss

**Figure 9:**
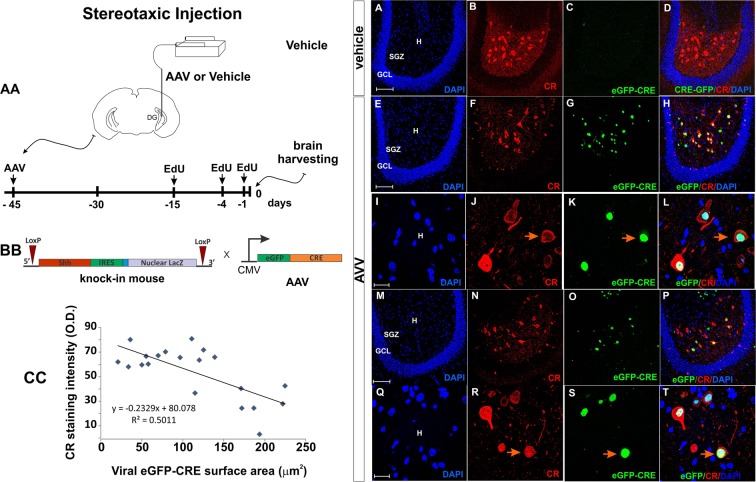
Ablation of Shh using an AAV virus. (**AA**) Experimental design. The e-GFP-CRE AAV virus or vehicle was injected into the hilar region on one hippocampus (left side). Histological observations were made 45 days after the injections. EdU was administered 15, 4 and 1 days before euthanasia (See methods). (**BB**) Constructs of the Shh-nlacZ Knock-in and in the AAV vector. The expression of the virus in target cells will induce deletion of the Shh gene. (**CC**) Plot correlating the degree of viral infection as measured by the surface area of nuclear eGFP signal with CR staining intensity shows that cells that have greater infection levels express less CR. (**A–H**) Low magnification comparison between vehicle side and AAV injected side, 45 days after the injections of the AAV virus in the DG of Shh-nlacZ mice. The expression of the virus can be seen in G and how the virus targeted the CR+ neurons can be seen in H (20X, bar 100 µm). (**I–L**) High magnification images showing that the infection leads to reduced CR expression and cell loss (100X, bar 20 µm). (For quantification of CR cells see Fig. 11R). (**M–P**) Other examples at low mag as in E-H (20X, bar 100 µm). (**Q–T**) Other examples at high mag as in I-L (100X, bar 20 µm). Abbr: GCL, granular cell layer; SGZ subgranular zone; and H, hilus.

**Figure 10:**
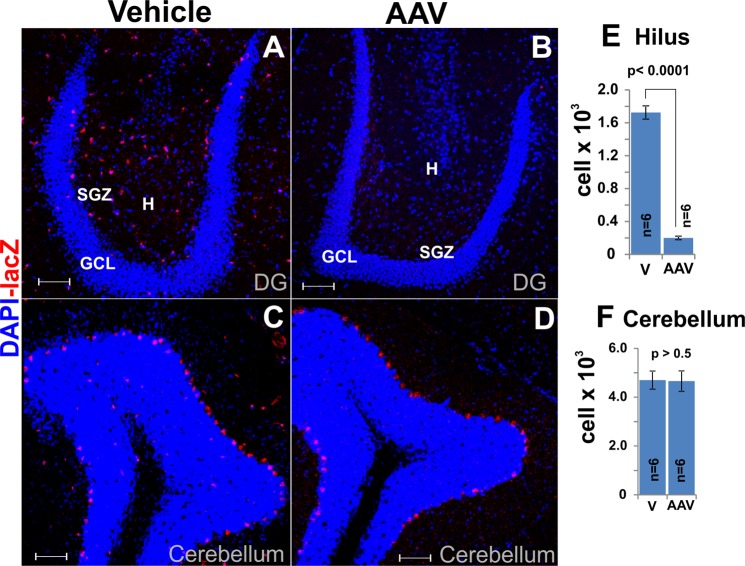
Shh-nlacZ expression was selectively suppressed in the hilus in the AAV-injected side but unaffected in cerebellum Purkinje cells. (**A,B**) Shh-nlacZ tracer expression in the DG in the vehicle (A) and AAV-injected side (B) (20X, bar 100 m). The expression of lacZ was almost completely abolished in the injection site. (**C,D**) Shh-nlacZ tracer expression in the cerebellum in the vehicle (C) and AAV- injected (D) side (20X, bar 100 m). Note that Shh-nlacZ expression in the cerebellum was not affected by the virus injection in the DG. (**E**) Quantification of the number of cells positive for the Shh-nlacZ tracer in the DG (unpaired, two tailed t-test). (**F**) Quantification of number of cells positive for the Shh-nlacZ tracer in the cerebelum (unpaired, two tailed t-test). Abbr: GCL, granular cell layer; SGZ subgranular zone; and h, hilus.

**Figure 11:**
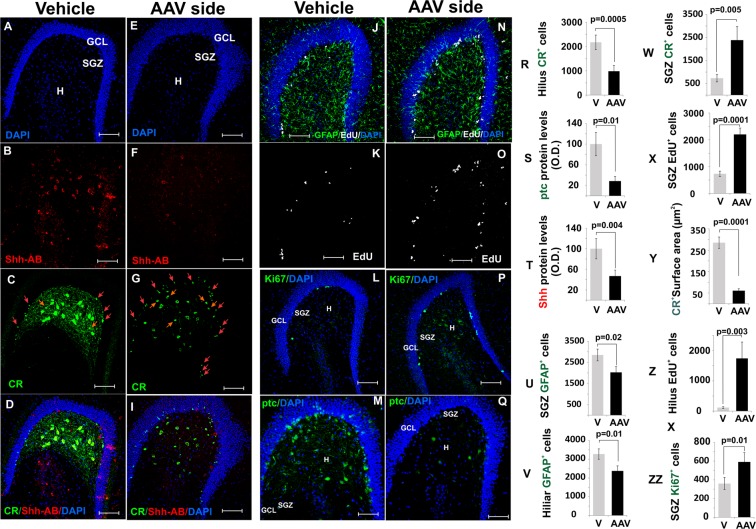
Effects of the Shh ablation on calretinin^+^ cells and proliferative markers. (20X, bar 100 um). (**A–D**) The vehicle-injected hippocampus show normal expression of Shh and CR proteins as detected by the antibodies (20X, bar 100 µm). (**E–I**) The AAV-injected hippocampus shows a substantial decrease of Shh levels and a striking reduction in size and numbers of CR+ cells. (20X, bar 100 µm). Also note the increase in small CR+ cells in the hilus (red arrows) and toward the hilus (yellow arrows) (see also **T,W**). (**J–K**) The vehicle-injected hippocampus shows normal expression of GFAP (glial cells) and EdU (20X, bar 100 µm). (**N,O**) The AAV-injected DG shows increased expression of EdU and a slight decrease in GFAP signaling (20X, bar 100 µm). (**L,P**) Ki67 also was found to be elevated in the AVV injected hilus (20X, bar 100 µm). (**M,Q**) Ptc protein expression decreased significantly followed the virus transfection (20X, bar 100 µm). (**R–ZZ**) Quantification of cells number and protein levels identify in the pictures above. The p scores correspond to unpaired (two tailed) t-test (n/group = 6). Abbr: GCL, granular cell layer; SGZ subgranular zone; and H, hilus.

To test whether *Shh* was necessary for the survival of CR neurons, we injected an *AAV9-eGFP-Cre* virus into the DG of *Shh*-*nlacZ* mice to induce the ablation of the conditional *Shh-nlacZ* allele. (Fig. 9AA,BB). In this mouse line, Cre activity removes exons 2 and 3 of the *Shh* gene and the *nlacZ* marker producing a *Shh* null allele and allowing for convenient identification and quantification of cells with ablated *Shh*. At day 45 following virus injection, animals were euthanized, and their brains perfused for histological analysis. Quantification of viral expressed GFP revealed that the virus was expressed in CR^+^ cells in the central hilus (Fig. 9G,H,K,L). We observed a reduction in CR staining intensity inversely proportional to GFP expression (Fig. 9CC.G–T). The expression of *nlacZ* in the DG was almost completely abolished among infected cells as compared to controls (Fig. 10A,B and E), while *nlacZ* expression in the cerebellum was unchanged (Fig. 10C,D and F), revealing the anatomical selectivity and efficiency of the viral infection mediated ablation of *Shh*. As expected, there was a marked reduction of the *Shh*-protein detected by immunohistochemical staining in the AAV injected mice compared to control mice injected with vehicle in the same hemisphere (Fig. 11B,F and T). Further, and consistent with reduced *Shh* signaling strength, we found that the expression level of *Ptc* protein, a transcriptional target of *Shh* signaling^31^, was downregulated by 80% (Fig. 11M,Q and S). Quantification of CR^+^ cells showed a pronounced, 60%, reduction in CR^+^ cell numbers (Fig. 11C,G and R). The average surface area of hilar CR^+^ cells was also drastically reduced from 285.2 µm^2^ to 60.8 µm^2^ (Fig. 11Y). Taken together, these results indicate that viral Cre mediated ablation of *Shh* abolished most of the hilar *Shh* signaling, which, in turn, critically compromised survival and/or marker expression of hilar CR^+^ cells.

### Ablation of Shh in CR^+^ neurons results in increased SGZ proliferation and neurogenesis

**Figure 12:**
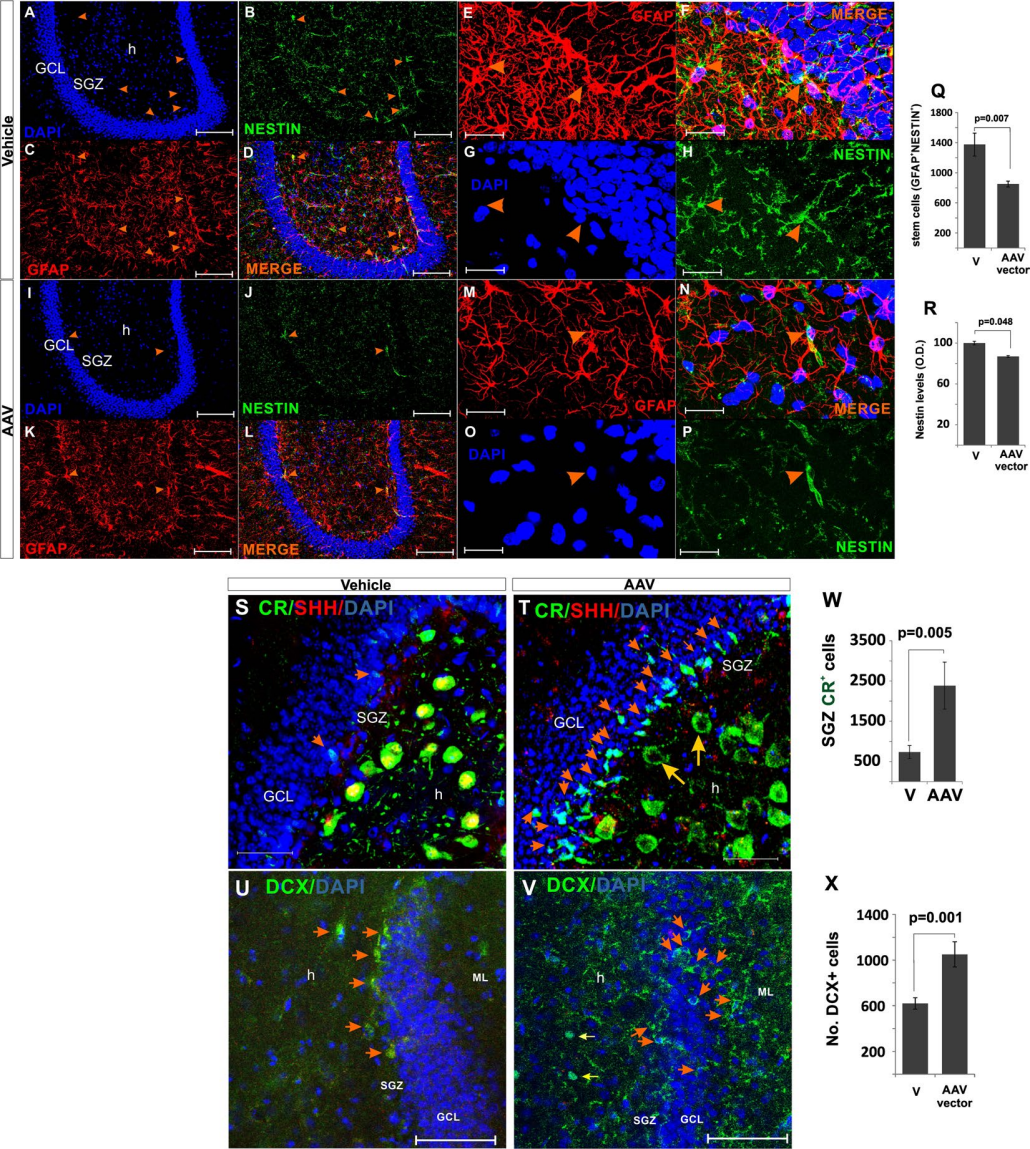
Effects of the Shh ablation on the SGZ radial glia stem cells (NSCs) population and immature neurons. (**A–D**) Double labeled GFAP+ Nestin+ cells (radial glia NSCs) as observed in the vehicle injected side (20X, bar 100 µm). (**E–H**) High magnification showing some GFAP+ Nestin+ cells from A-D (100X, bar 20 µm). (**I–L**) Panoramic view showing that the number of radial glia NSCs was reduced in the AAV-injected DG (20X, bar 100 µm). (**M–P**) High magnification from I-L (100X, bar 10 µm). (**Q**) NSCs were reduced in the AAV-injected DG by 40%. (**R**) Nestin protein levels slightly dropped in the injected side. (**S,T**) Shh knock-down produced a substantial increase in small CR+ cells in the SGZ (orange arrows). Note the lack of Shh protein in CR+ cells in the AAV group in T (yellow arrows). (**U,V**) Shh knock-down also upregulate DCX signaling, increase in number of cells in the SGZ migrating to the ML (**W–X**) Quantification of small SGZ CR+ neurons (W) and DCX+ cells reveals increased neurogenesis in the AAV injected site. The p scores correspond to unpaired (two tailed) t-Test (n/group = 6). Abbr: GCL, granular cell layer; ML, molecular layer; SGZ, subgranular zone; and h, hilus.

We next tested whether the ablation of *Shh* from hilar MCs would impact neurogenesis. Contrary to our expectations, the decrease in *Shh* expression in the hilus *increased* SGZ proliferation as shown by EdU incorporation (Fig. 11K,O and X) and Ki67 staining (Fig. 11L,P and ZZ). Quantification of GFAP^+^ cells in the hilus shows a moderate decrease in the number of GFAP^+^ cells in the virus-injected hilus leading to altered GFAP^+^ scaffolding in the SGZ (Fig. 11J,N and V) and reduced numbers of GFAP^+^, EdU^+^ NSCs (Fig. 12A–P and Q). Importantly, there was an increase in the number of small CR^+^ cells located in the SGZ (Fig. 12S,T and W). These small triangular CR^+^ cells in the SGZ and in the inner granular cell layer have been described as new born or immature neurons that transitorily express calretinin during maturation^32,33^. Consistently, we found an increase in the numbers of DCX^+^ cells (Fig. 12U,V and X), a marker for immature neurons^34^. Noteworthy, these newly formed CR^+^ and DCX^+^ cells were mostly located towards the granular cell layer (GCL) and the molecular layer (ML) (Fig. 12T,V) forming a pattern distinctly different from the increase in hilar CR^+^ cells observed during KA-induced epileptiform activity (Fig. 5B). Together, we find that the hilar ablation of *Shh* results in an increased production of neuronal precursor cells that migrate preferentially into the GCL while the KA induced up regulation of *Shh* expression is associated with migration of immature CR^+^ cells into the hilus from the GCL.

## Discussion

Here we identified hilar MCs as a prominent source of *Shh* within the adult hippocampus. Our data indicates that *Shh* signaling originating from MCs is critical for their survival upon excitotoxic insults and the modulation of dentate gyrus neurogenesis.

**Figure 13:**
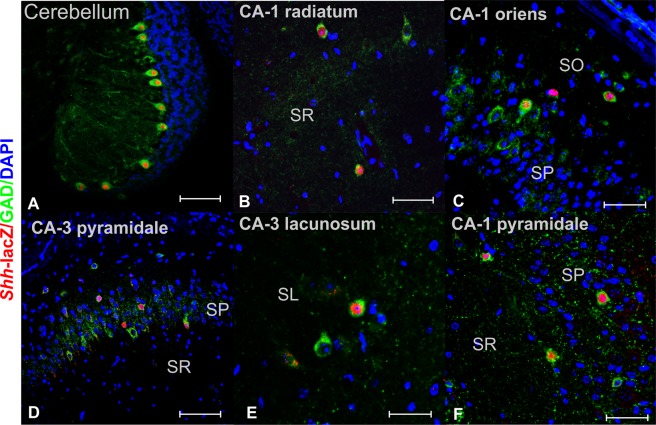
Shh-nlacZ expression in the hippocampus and cerebellum was restricted to GABAergic interneurons. (**A**) Panoramic view of cerebellum folia showing the expression of Shh-nlacZ in GABAergic Purkinje neurons (25X, bar 100 µm). (**B–F**) Overall view of CA1 and CA3 regions of the hippocampus showing expression of Shh-lacZ restricted to sparse GABAergic cells. The cellular sub-type was not CR, PV, NPY or STT positive (remains unknown). All imagines were 25X (B and D bar = 100 µm) (C, E and F bar = 50 µm). Abbr: SR, stratum radiatum; SP, stratum pyramidale; SO, stratum oriens; SL Stratum lacunosum; CA1, Cornu Ammonis-1; CA3, Cornu Ammonis- 3.

These studies were enabled by a sensitive gene expression tracer allele for *Shh* which was produced by homologous recombination resulting in the continuous bicistronic transcription of *Shh* and *nlacZ* genes under the control of the endogenous *Shh* promoter^13^. The faithfulness of this recombinant allele during development and in the adult brain was extensively verified^13,35–40^. A recent study by Ortega *et al*.^41^ confirms the pattern of *Shh* expression from the wt allele and from the gene expression tracer allele in the nigro-striatal system. Likewise, in the cerebellum, *Shh*-*nlacZ* was consistently expressed in large GABAergic neurons known as Purkinje cells (Fig. 13A), in agreement with previous reports^42^, further corroborating the fidelity of the *Shh*-*nlacZ* gene expression tracer allele. Using this gene expression tracer allele we found that among *Shh*-*nlacZ*^+^ cells in the hilus, more than 90% co-expressed calretinin (CR) and GluR 2/3, the neurochemical signature of mossy cells. Among all CR^+^ neurons, only the large multipolar neurons in the hilus, which traditionally have been considered mossy cells^14^, expressed *Shh*.

Mossy cells are the only excitatory cell type in the hippocampus that can be considered a type of “feedback interneuron”^43^. In fact, some authors have cataloged MCs as excitatory interneurons^44,45^. As an interneuron, a single MC receives convergent inputs from granular cells through mossy fibers, sending back short but highly distributed signals to a proportionally greater number of granule cells. We also show here that MCs express CR and innervate profusely other MCs – displaying a pattern of short reciprocal MC to MC projections (Fig. 7B). These results agree with previous EM findings^46^, and are in line with the finding that reciprocal connections are a distinctive feature of calretinin + neurons^30^. While there is no conclusive proof for Mossy to Mossy cell innervation, our data are consistent with results from Wenzel *et al*.^46^ who argued for cross innervation by collaterals. Although many MCs extend their projection to the DG inner molecular layer, MCs form a plexus of exuberant axon collaterals within the hilus, which has been recognized in anatomical^47^ and electrophysiological^16,48,49^ studies. These results also show that MCs are immunoreactive to GABA and GAD proteins, which does not necessarily imply that the cells are functionally GABAergic but, rather that mossy cells express GABA like other glutamatergic neurons^50–52^.

The neural cell damage in *Shh*-expressing cells and massive cell loss induced by *Shh* ablation revealed that *Shh* expression is critical for the long-term survival of MCs. Conversely, *Shh*^+^ signaling was increased during the KA-induced hyperactivity. This upregulation of *Shh* and a concomitant increased expression of the *Shh* effector *Smo*, conferred a survival advantage to CR^+^ neurons expressing *Shh*, since these cells increased in numbers. Exaggerated neural activity, inflammation and persistent oxidative stress may lead to *Shh*/*Smo* upregulation as observed during amphetamine treatment^53^, facial nerve axotomy^54^, hypoxia^55^ or ischemic stroke^56^. Nevertheless, the upregulation of *Shh* during KA-induced neural hyperactivity did not seem to affect SGZ proliferation even when *Smo* signal was upregulated in the SGZ (Fig. 8B,K). Bragina *et al*.^57^ showed that rising *Shh* signaling by increasing *Smo* agonist (SAG) concentrations from 1 nM to 5 nM decreased proliferation from significantly high levels (at 1 nM) back to control levels (at 5 nM) in hippocampal cell cultures. Further, when *Shh* concentrations increased from 10 nM to 50 nM significant cell death was observed in a cerebellar primary culture. Thus, it is possible that *Shh*’s effects on proliferation *in vivo* are modulated in narrow time dependent concentration ranges. *Shh* may be a factor that maintains or stimulates proliferation at low concentration levels but an increase over some concentration limits or for longer periods of time may lead to inhibition.

The association between Shh upregulation and decreased -instead of increased-neurogenesis agree with previous studies in the SVZ. Thus, activation of the Shh pathway, through the conditional ablation of *Ptc*, led to a dramatic decrease in neurogenesis in the SVZ. The neurogenesis blockade was related to a shift in NSC division mode from asymmetric to symmetric^58^. Sustained activation of the *Shh* pathway using this strategy also induced a progressive increase in NSC numbers in the quiescent state and a marked reduction of the activated NSC pool, leading to an almost complete exhaustion of neurogenesis^59^. Our results in the SGZ agree with the studies in the SVZ. Collectively, these studies suggest that the response of *Shh*-producing neurons is adapted to preserve NSCs under changing conditions in the neurogenic niches. Under normal conditions, basal levels of *Shh* would act as a trophic agent for quiescent-NSCs allowing them to homeostatic be converted to activated-NCSs and increasing the production of intermediary progenitors. Under excitotoxic conditions, *Shh* is upregulated (e.g. by mossy cells in the hilus), which lead to inhibition of the NSCs activation process and a decline in neurogenesis. This produces the accumulation of quiescent NSCs^59^. Quiescent NSCs are more resistant to toxic conditions because replicate slowly^58^. The consequence of this *Shh*-concentration dependent effect is that NSCs are preserved in the neurogenic niches.

During the second week post KA-injection we observed a hyperexcitability syndrome characterized by stereotypes, forelimb clonus (Racine’s stage 3) and electrographic abnormalities such as high-amplitude bursting and hyper-synchronized spiking. During this epileptogenic process, the MCs survived and there was an upregulation of *Shh* and *Smo*. In more severe epilepsy, i.e. when KA is directly administered into the DG and epilepsy stages 4/5 become persistent, MC death might occur, in which case, a fall in *Shh* levels as well as an increase in neurogenesis could be expected^60^. However, while some authors have considered MCs “vulnerable”, others find that the loss of both MCs and interneurons is equivalent in epilepsy^61^. Furthermore, in humans with epilepsy, Seress *et al*.^62^ found that MCs were present in the hilus of the DG when most pyramidal neurons of the CA1 and CA3 areas were lost.

The neurogenic response to neurotoxic insults is biphasic: the immediate response is an increase in neurogenesis which is followed, after a variable time span, dependent on experimental conditions and species, by a decline in neurogenesis. Low levels of proliferation and neurogenesis persist chronically. In the KA model, several groups using intracerebral KA administration have showed that proliferation increases during the first 3 days after the KA injections. By the end of the first week after the injection, there is a fall in the levels of SGZ proliferation. After 7 days of the injection, the levels of proliferation/neurogenesis are normal or reduced^26–28,60^, while a deficiency in proliferation and neurogenesis persist chronically^26,27,60^. In addition, the initial increase in proliferation after the KA injection have been associated with expansion of the glial pool rather than the neuronal pool^26,60^. In these KA-studies, *Shh* levels were not measured at different stages of the experiments and further investigations are required to establish whether there is a consistent relationship between *Shh* levels and neurogenesis in this model.

Conversely, the dramatic reduction in *Shh* protein levels after the ablation of the *Shh* gene leads to neurodegeneration, atrophy and death of MCs and to an increased proliferation and neurogenesis in the SGZ. Thus, in the SGZ adjacent to hilar CR cells, where the ablation took place, we found significant increases in EdU, Ki67 (Fig. 11), and immature neuron markers (CR and DCX) in the SGZ (Fig. 12). The number of GFAP^+^EdU^+^ radial glia cells were diminished, and this could be interpreted as a depletion of NSCs due to the massive and prolonged proliferative response^60^ (Fig. 12). Interestingly, chronic ablation of MCs transiently increases the activation of NSCs (at 15 days after ablation), leading to a NSCs depletion at 42 days after ablation^20^, which supports the present finding that NSCs are depleted 45 days after the initial CRE induced gene ablation.

In the SVZ of the adult brain, it has been shown that *Shh* exerts both positive and negative regulatory actions on neurogenesis through *Smo* and *Gli3*, respectively. NSC proliferation and neurogenesis appear to be dominated in the SVZ by *Gli3R* repressor activity. Thus, removing *Gli3* in *Smo* conditional mutants largely rescues neurogenesis, while, expression of a constitutive *GLI3(R)* abrogates neurogenesis^63^. If a similar signaling mechanism would take place in the SGZ, the downregulation of *Shh* could lead to decreased repressor activity and increased proliferation. Molecular interactions of this type may help explain why the effects of a *Shh* deletion (in *Shh* sources) may greatly differ from the effects of a *Smo* deletion (in *Shh* target cells)^6^.

The present studies are the first to identify the cells that synthetize *Shh* in the ventral adult hippocampus, an area where most MCs are located^14^. From horizontal brain section, we visualized the areas where the hippocampus was cross sectioned to accurately localize DG sub-regions (see Fig. 1A and methods). Li *et al*.^6^, using a *Shh*-gfpcre transgenic, followed the *Shh* lineage and found that CR neurons were actively producing *Shh* in the hippocampus at P15. MCs were recognized as CR^+^ cells in the dorsal but not the ventral DG at P15. However, these authors did not assess whether these putative MCs were positive for either GABA or glutamate markers, thus leaving their identity ambiguous. Several authors have found that mossy cells are CR^+^ only in the ventral DG and not in the dorsal DG^14,25,32^, while at dorsal DG levels, calretinin immunoreactivity was limited largely to a subpopulation of interneurons^64^. Liu *et al*.^32^ identified in the hilus many CR immunoreactive cells as GABAergic and some that express both GABA and glutamate markers. Our results show that cells expressing CR, GluR2/3 or CGRP also co-stained for α-GAD and α-GABA antibodies. Among lacZ^+^ cells, over 90% were positive for GAD, CR, GluR2/3 or CGRP (Table 1, columm-7). Further, a high GABA-CR colocalization can be observed in double *in situ* hybridization (FISH) images from 2 mice (P57) published in the Allan Atlas^21^. From these images, we estimated that among GAD-1^+^ cells, about 70% were positive for CR (Fig. 3B). These observations support our finding using IHC that most hilar CR^+^ cells in the ventral hilus are GAD1^+^ (GAD67) (Figs 4 and 5). There are many factors related to IHC technique that can account for the discrepancy with other groups. An advantage of our methodology was the strong, selectively nuclear, immune-reactivity for lacZ, These lacZ^+^ cells were immunoreactive within the cytoplasm to α-GAD antibodies at conventional concentrations (1:500 to 1:1000) and using several commercial brands including α-GAD65/67 (ABcam), α-GAD67 and α-GAD65 (Santa Cruz) and α-GABA (Sigma-Aldrich). In addition, we showed that MCs expressing *nlacZ* strongly co-localized with GluR2/3 (Fig. 3C,G) and CGRP (Supplemental Fig. S2). GluR2/3 and CGRP cytoplasmic signals highly co-localized with GABA markers in the hilus (Fig. 3D–F,H; Supplemental Fig. S2). Collectively, these observations suggest that MCs express both glutamatergic and GABAergic markers like medium spiny neurons (MSNs) in the striatum^65^ or the granular cells in the dentate gyrus^50^.

In addition to the well-known effects of *Shh* on NSCs in the SGZ, *Shh* could be delivered to the GCL via axonal transport by *Shh* expressing MCs and could influence cell differentiation/maturation and the continuous integration of granular cells produced by neurogenesis into the hippocampal circuits. MCs have recently been implicated in place fields neural processing^66^, spatial exploration and pattern separation^67^ and in generating a special type of LTP^68^. Our findings will prompt follow up studies that will seek to clarify whether hippocampal learning and memory processes might depend on *Shh* expression by MCs and whether *Shh* might regulate structural plasticity in hippocampal circuitry.

In conclusion, we have found that *Shh* expression in MCs is dynamic and can be upregulated during epileptogenesis to enhance survival. Furthermore, we show that *Shh* expression by MCs is indispensable for their survival. Finally, *Shh* ablation led to reactive proliferation of immature neurons and depletion of NSCs suggesting that MC derived *Shh* has multiple regulatory functions during neurogenesis. As MCs are implicated in learning and memory, the modulation of *Shh* could be a key strategy to modulate plasticity and neurogenesis. This suggests that the modulation of *Shh* expression in MCs might be a potential approach to generate therapies aimed at protecting neural circuits in epilepsy and excitotoxicity and treating cognitive and emotional disorders in which altered neurogenesis is a major component.

## Methods

### Mouse strains

**The** ***Shh*-*nlacZ*** **allele** was generated by homologous recombination in ES cells by Dr. A. Kottmann and T. Jessell. Additional details of the allele construction are described in Gonzalez-Reyes *et al*.^13^.

Transgenic mice expressing the oligodendrocyte reporter *PLP*-EGFP were generated by Dr. W. Macklin and described in Mallon *et al*.^69^.

**CAG-H2B-EGFP (**B6.Cg-Tg(HIST1H2BB/EGFP)1 Pa/J) were obtained from Jackson Laboratory (stock number: 006069).

Animals were housed no more than 5 adult animals per cage and maintained in a SPF room under light (12-h light/12-h dark cycle), temperature and humidity controlled conditions. For acute *in vivo* experiments, adult male animals were used (range from P60 to P90). All experimental procedures performed in this study followed the NIH animal use guidelines and were approved by the Institutional Animal Care and Use Committee (IACUC) at Case Western Reserve University.

### KA injections

The dose of KA was fractioned (5 mg/kg IP every 30 min) until a maximum of 35 mg/kg; mice were scored using a Racine’s severity scale^24^. Only mice that reach status epilepticus during administration of KA were selected for histological studies. The histological studies were performed 2 weeks after the KA injections.

### BRDU injections

Two doses of BRDU (30 mg/kg each) separated by three hours-intervals were injected (IP) seven days before euthanasia. BRDU staining procedures are described below.

### Histological procedures

For histological studies, knock-in and transgenic mice or littermate wild type mice were transcardially perfused with ice-cold 4% paraformaldehyde (PFA) under isoflurane anesthesia. Brains were then removed, post-fixed in 4% PFA at 4 °C overnight, and cryoprotected in 30% sucrose in phosphate-buffered saline (PBS) for 2 days before being flash-frozen in 2-methylbutane on dry ice and stored at − 80 °C. Brains were then sectioned at a thickness of 40 μm using a cryostat (Leica CM3050S), and free-floating sections were blocked in 5% horse serum for 1 h, then stained for α-GAD-65 (Santa Cruz sc-32270, 1:1000 dilution), α-GAD-67 (Santa Cruz sc-5602, 1:700), α-GAD-65/67 (abcam, ab49832, 1:1000), α-GABA (Sigma, A2052, 1:500), α-parvalbumin (abcam, ab11427, 1:1000), α-somatostatin (abcam, ab8904, 1:1000), α-nestin (abcam, ab6142, 1:1000), α-*Smo* (abcam, ab80683, 1:500), α-sonic hedgehog (abcam, ab50515, 1:1000), α-NeuN (abcam, ab131624, 1:1000), α–calretinin (abcam, ab702, 1:400), α-neuropeptide Y (abcam, ab30914, 1:1000), α-beta Galactosidase (abcam, ab936, 1:1000), α-*Ptc*-1 (abcam, ab53715, 1:1000), α-GFAP antibody (abcam, ab7260, 1:1000; Sigma-Aldrich, 1:200), α-GluR2/3 (abcam ab6438, 1:250; Millipore AB1553, 1:250), α-GluR2 (Santa Cruz 517265, 1:200), α-CGRP (ab36001, 1:500), α-doblecortin (Santa Cruz, sc-271390, 1:500) and α-BRDU (abcam ab6326, 1:200) at 4 °C for 24–36 h. The specificity of α-CGRP antibody, abcam ab36001, was validated by Carter *et al*.^70^ in neurons of the parabrachial nucleus (PBN) in mouse brainstem. Sections were then incubated with secondary antibodies conjugated to Cy3 (1:700, Jackson ImmunoResearch) or Alexa Fluor 594 or 488 antibodies (1:1000, Life Technologies). After rinsing in PBS, sections were mounted on Fisherbrand superfrost/plus microscope slides in vectashield mounting media (Vector Laboratories, Burlingame, CA). DAPI (1:3000, Invitrogen) was included during secondary antibodies incubation to visualize nuclei.

For BRDU staining the procedure was the same as above but the slices were pretreated with citrate buffer and heated to 99 °C in a water bath before blocking^71^. Chromogenic immunohistochemistry was performed using the above-mentioned primary antibodies and developed using betazoid DAB, vulcan fast red and hematoxylin (BioCare Medical Kit).

Cells were quantified from 10 horizontal sections of 40 µm thickness each that were spaced 160 μm apart (4-section interval) covering the entire the dorsal-ventral- and anterior to posterior- extent of the hilus. The horizontal slices allowed to sample the volume represented in Fig. 1. The number of cells was estimated using a stereological approach^13^ by two independent observers blinded to the experimental groups. Fluorescent colocalization was demonstrated using Z-stack reconstructions of the whole cell allowing orthogonal views at 63X (Zeiss LSM 510 microscope) or 100X (Olympus FV-1000 microscope) magnification. Cells were counted exhaustively from images with a full view of the hilar region (25X). To count double-labeled cells we took Z-stacks at 1μm-steps across the section and generate a Z-projection (vertically superimposed) into a single x-y plane (FV10-ASW software). The projected image was electronically amplified 4 times (to 100X) on the computer screen. Using the image J cell-counting tool only cells with clear morphology and delineated nucleus were counted and marked to avoid repetitions. Co-localization was reported as average ± SEM of cells that co-express two markers for every 100 cells expressing one of the markers (Table 1). To determine optical density, the channel corresponding to the protein was selected. Using image J, the integrated optical density (IOD) and the cell surface area (CSA) were measured and the IOD/CSA ratio was calculated for each immune positive cell. This value was then average and normalized to controls.

### AAV virus and EdU experiments

*Shh*-*nlacZ* animals (P50, n = 6/group) were stereotaxically injected with an AAV virus (AAV9.CMV.HI.eGFP-Cre.wPRE.SV40) or vehicle to the DG hippocampus (bregma -3.3 mm, lateral 2.7 mm and ventral -4.2 mm, left hippocampus)^72^ and euthanized 8 weeks later. The injections volume was 0.5 µl at the rate of 0.1 µl/min. Animals received 3 IP injections of EdU (50 mg/kg each) 4 hours apart at days -15, -4 and -1 before euthanasia (at P95) (See Fig. 9AA). At the end of the experiment, animals were perfused transcardially with PFA 4%, the brains were dissected out and processed for cryostat sectioning (40 µm). EdU was stained using the Click-It method (Invitrogen).

### Data analysis and statistics

Student’s t-tests were used to compare either two repeated (*paired-t-test*) or independent (*unpaired-t-test*) measures. Test results and number of animals per group are reported in the figures or in the figure legends. A statistical significance criterion of α = 0.05 was used for all tests. Results are shown as mean ± standard error (SEM). Statistical test results and group sizes are reported in the figure legends.

## Supplementary information

Supplementary Dataset 1
