# Supplementary material for: Sonic Hedgehog is expressed by hilar mossy cells and regulates cellular survival and neurogenesis in the adult hippocampus
Source: Sci Rep. 2019 Nov 22;9:17402. doi: 10.1038/s41598-019-53192-4 (PMC6874678; doi:10.1038/s41598-019-53192-4)
Supplement: Supplementary file 1 — Supplementary Dataset 1 [file 41598_2019_53192_MOESM1_ESM.pdf]

Supplementary Material

Sonic Hedgehog is expressed by hilar mossy cells and regulates cellular survival and neurogenesis in the adult hippocampus

Luis E. Gonzalez Reyes, Chia-Chu Chiang, Mingming Zhang, Joshua Johnson, Manuel Arrillaga Tamez, Nicholas Couturier, Neha Reddy, Lev Starikov, Jeffrey R. Capadona, Andreas H. Kottmann, and Dominique M. Durand.

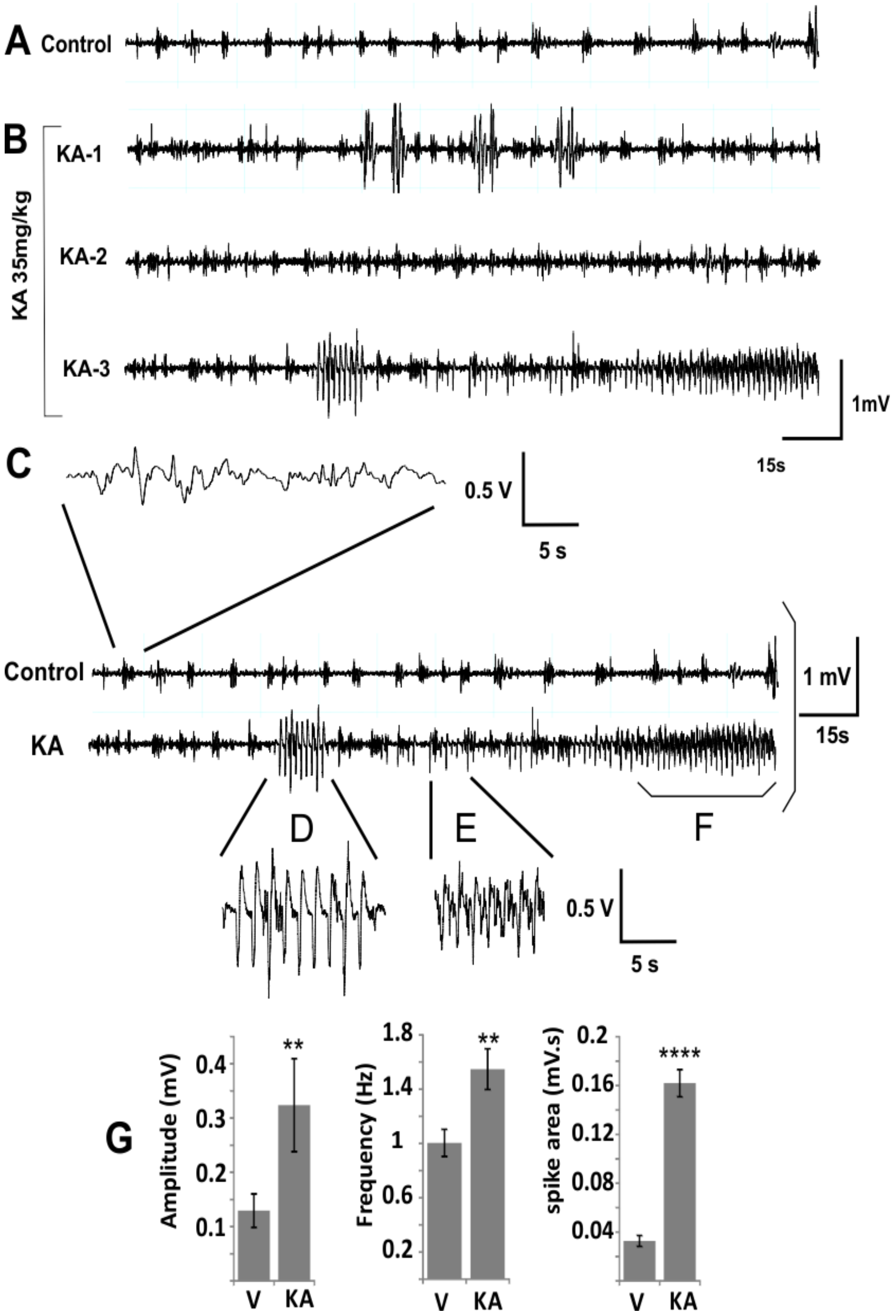

Supplementary Fig. 1S. Epileptiform activity is induced in KA-injected hippocampus

Local field potentials (LFP) in the CA1 region of the right hippocampus were recorded during periods of quiet rest to avoid motion artifacts.

A. Representative traces from a control animal CA1 region, which show spontaneous oscillatory activity further described in C. (See Supplementary Methods for information on the *in vivo* recording procedures and parameters).

B. Representative traces from KA injected animals show apparent increase in spike frequency and amplitude. KA injected mice seem to have lost the normal pattern of oscillatory activity showing random bursts of activity with exaggerated spike amplitude or hyper-synchrony.
C. Rhythmic burs of theta activity in control mice during resting (quiet) state (non-sleeping animal lying on the cage floor).
D. Typical interictal activity that appears here as synchronized spikes (~1 Hz) in KA-injected mice during resting periods recorded on day 14 after KA injection.
E. Neural firing could progress to hyper synchronized firing observed during quiet rest periods. Behaviorally, some animals showed clonus or oscillatory shaking grade 3 Racine scale). However, neither electrographic seizures nor full blown behavioral seizures (Rearing and falling with forelimb clonus) were observed during several hours of observation on the second week after KA injection (see Fig. 3C).
F. KA injected animals showed increased amplitude, frequency and spike area as recording in the CA1 pyramidal cells. Data are average/30 min on day 14 after KA injection. ** $p<0.001$, ****$p <0.0001$, student t-test ($n=3$/group).

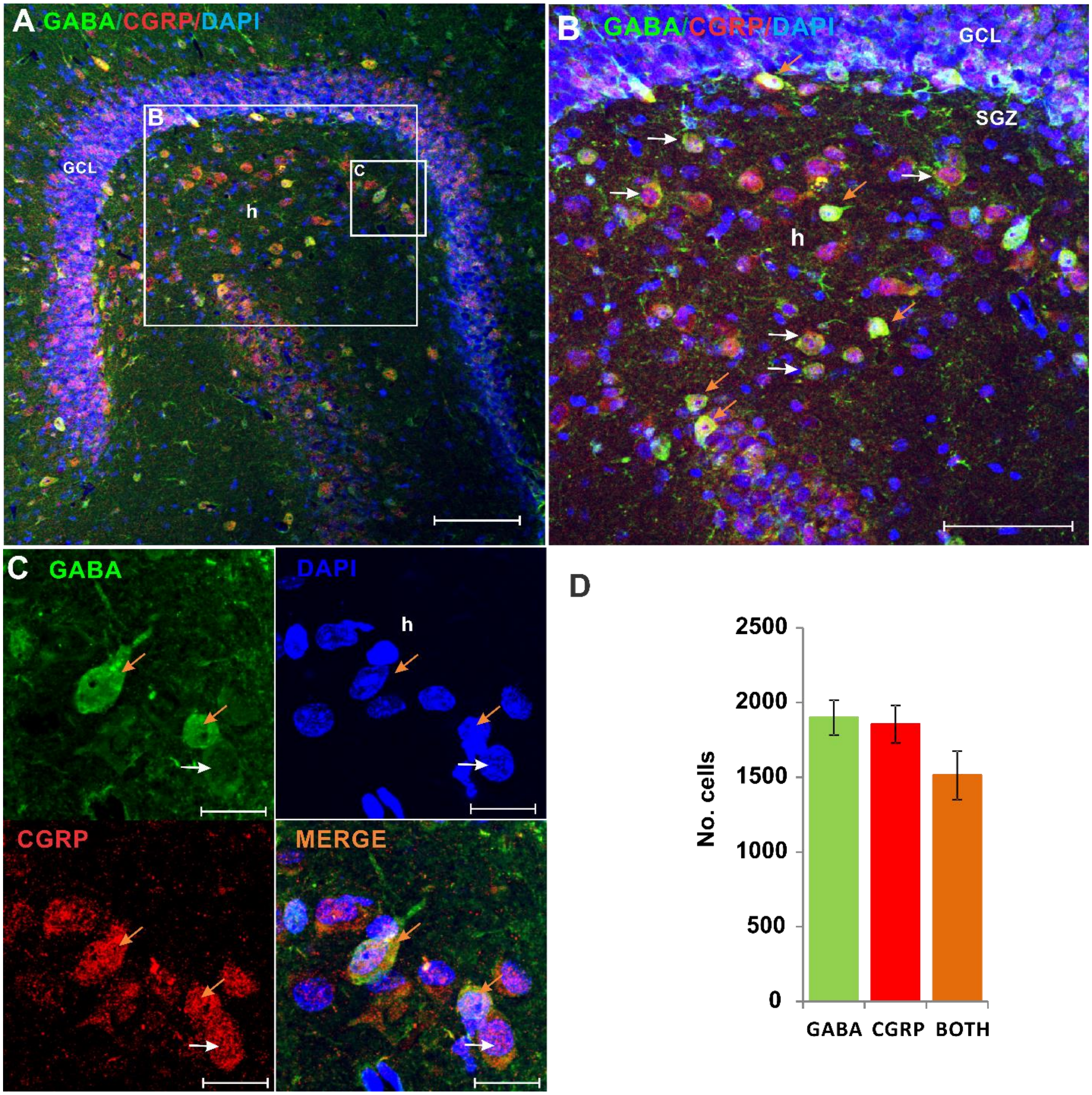

Supplemental Fig 2S. CGRP colocalized with GABA in hilar mossy cells

A. Panoramic view fluorescent IHC for calcitonin gene related peptide (CGRP) and GABA in the dentate gyrus (Bar=100µm).

B. Zoom into A (40X) to show high (orange arrow) and low (white arrow) levels of GABA staining in cell positive for both CGRP and GABA. (40X, bar=50µm)

C. Zoom into A (100X) IHC staining showing separated channels to confirm GABA-CGRP co-localization (arrows) (see quantification in Graph E below). (100X, Bar=20µm). Note the high (orange arrow) and low (white arrow) intensities of the GABA staining in different CGRP positive cells.

D. Analysis of the GABA and CGPR co-expression show that a 79.6 % of GABA+ cells colocalized with CGRP+ cells; while an 81.6% of CGRP+ cells colocalized with GABA+ cells (n=3mice x 10 slices).

Abbr: GCL, granular cell layer. SGZ sub-granular zone and h, hilus

Supplementary Methods:

***In vivo* local field potential (LPS) recording**

Stereotaxic surgery was performed under 3% isoflurane anesthesia. A Pt:Ir electrode (1cm long, 1mm insulated, 25µm diameter) was implanted into the CA1 right hippocampus (AP −1.3, L +2.0, DV −2.1mm) to record neural activity. Two screw electrodes were positioned in the skull and used as reference electrode (parietal location) and ground electrode (frontal location). Screw electrodes were placed in the skull for use as the reference (positioned caudally in the occipital bone) and system ground (positioned rostrally in the frontal/coronal bones). Animals received either vehicle or 35 mg/kg of KA on day 7 after surgery following the same procedure described with the group without implantation (see Methods). On day 14 after KA injection, LFP were recording in freely moving animals. Recorded neural activity was bandpass filtered (1 Hz to 5 kHz) and amplified (gain of 100, A-M Systems Model 1700 Differential AC Amplifier), then digitized at 20 kHz (PowerLabs/16SP) using LabChart8 software (ADInstruments, Dunedin, New Zealand) and stored for off-line analysis (Gonzalez Reyes et al., 2013; Ladas et al., 2015)

Supplementary References

1. Gonzalez-Reyes LE, Ladas TP, Chiang CC, Durand DM. TRPV1 antagonist capsazepine suppresses 4-AP-induced epileptiform activity in vitro and electrographic seizures in vivo. Exp Neurol. 2013;250:321-32.

2. Ladas TP, Chiang CC, Gonzalez-Reyes LE, Nowak T, Durand DM. Seizure reduction through interneuron-mediated entrainment using low frequency optical stimulation. Exp Neurol. 2015;269:120-32.
